# Supplementary material for: Genomic Comparison of Insect Gut Symbionts from Divergent Burkholderia Subclades
Source: Genes (Basel). 2020 Jul 3;11(7):744. doi: 10.3390/genes11070744 (PMC7397029; doi:10.3390/genes11070744)
Supplement: Supplementary file 1 [file genes-11-00744-s001.zip › Supplemental_Data_R1/Takeshita_and_Kikuchi_Genes_2020_Supplemental_Figure_R1.pdf]

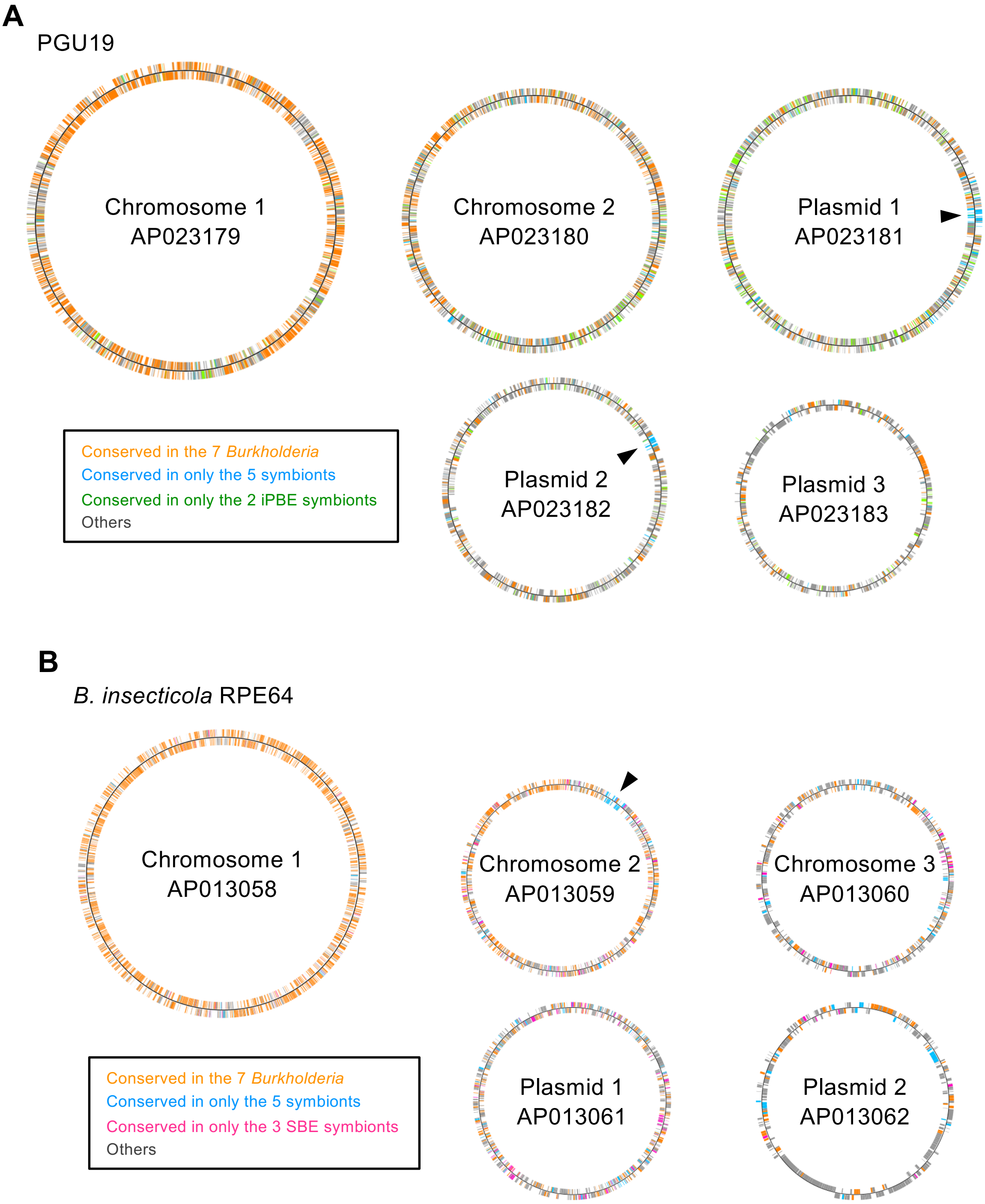

Figure S1. The distribution of the conserved genes on the genomes of the iPBE symbiont PGU19 (**A**) and the SBE symbiont *B. insecticola* RPE64 (**B**). The arrow head indicates the region that contains the cluster of conserved genes in the five insect symbionts.
